# Willingness of Chinese medical students to work on the frontline during a potential mpox outbreak: a nationwide cross-sectional study

**DOI:** 10.7189/jogh.16.04327

**Published:** 2026-11-06

**Authors:** Jiaxin Chen, Jie Ge, Yunfeng Han, Zhiping Xie, Honglong Li, Yan Zhao, Yuehui Jia

**Affiliations:** 1 School of Public Health, Qiqihar Medical University, Qiqihar, China; 2 School of Mental Health, Qiqihar Medical University, Qiqihar, China

**Keywords:** mpox, medical students, frontline work, willingness, public health emergency

## Abstract

**Background:**

The global mpox epidemic has been designated a public health emergency of international concern, placing substantial strain on public health systems worldwide. As a critical reserve workforce for outbreak response, medical students’ willingness to serve on monkeypox (mpox) frontlines remains understudied, and evidence on their mpox-related knowledge, attitudes, and practices and their association with frontline work willingness is still limited.

**Methods:**

We conducted an online nationwide cross-sectional survey among Chinese medical students from November 2023 to March 2024 using snowball sampling. We used multinomial logistic regression to identify independent influencing factors.

**Results:**

We included a total of 4,098 participants. Overall, 77.06% were willing to perform frontline mpox work, 18.57% were hesitant, and 4.37% refused. Among participant characteristics, studying medicine at others’ suggestion was associated with both higher hesitancy (odds ratio (OR) = 1.425; 95% confidence interval (CI) = 1.142–1.776) and refusal (OR = 1.635; 95% CI = 1.110–2.407). Greater mpox knowledge (OR = 0.973; 95% CI = 0.966–0.981) and higher vaccination-related behaviour scores (OR = 0.894; 95% CI = 0.882–0.907) were associated with lower hesitancy to work on the mpox frontline. Higher vaccination-related behaviour scores were also associated with lower refusal (OR = 0.883; 95% CI = 0.862–0.904).

**Conclusions:**

Most Chinese medical students demonstrated favourable willingness to support frontline mpox response. Medical education should strengthen training in mpox prevention and professional ethics. Public health policies should use risk-stratified deployment, provide adequate protection and psychological support, and establish targeted mobilisation mechanisms to improve the emergency response capacity of the medical student reserve workforce.

Monkeypox (mpox) virus was first isolated in 1958 [1,2], and remained a neglected tropical disease confined to Central and West Africa for decades following the first documented human case in 1970 [3]. Outbreaks in endemic regions were primarily driven by zoonotic transmission before 2022, with limited human-to-human spread [4]. This long-standing endemic pattern contributed to insufficient global preparedness for large-scale cross-regional transmission. A paradigm shift occurred in May 2022, when sustained community transmission was reported across multiple non-endemic regions without travel links to endemic areas [5]. This unprecedented global mpox outbreak prompted the World Health Organization (WHO) to declare a public health emergency of international concern in July 2022 and reaffirmed the declaration on 14 August 2024 [6,7]. This shift raised renewed concerns about pandemic potential and public health system preparedness. From 1 January 2022 to 28 February 2026, the WHO had confirmed 181,164 cases across 144 countries [8]. The first imported mpox case was reported in Chongqing, China, in September 2022, and an imported case was reported in June 2023 [9]. Mpox was officially included in the management of Class B infectious diseases in China in September 2023 [10]. Therefore, a large mpox outbreak could place substantial pressure on the healthcare system and exacerbate shortages of healthcare resources.

Frontline medical and public health personnel are the core of epidemic response, yet they face elevated occupational risks and widespread work-related burnout during public health emergencies [11]. Previous studies have also reported considerable reluctance or hesitancy among healthcare workers to participate in epidemic response work. For example, one survey found that 35.9% of frontline healthcare workers were unwilling to work during the COVID-19 pandemic, whereas another reported that 42% of participants were less willing to work under pandemic conditions than before the pandemic [12,13]. A systematic review and meta-analysis further indicated that healthcare workers’ willingness to work during an influenza pandemic varied widely, ranging from 23.1% to 95.8% [14]. Against this background, medical students, as a reserve component of the future healthcare workforce, have become an important supplementary resource in emergency response. Beyond alleviating staffing shortages during peak epidemic periods, their willingness to engage in frontline work also reflects core professional qualities, including medical ethics, social responsibility, and professional identity. These factors are closely related to their career development and may influence the long-term stability and emergency response capacity of the healthcare workforce. Existing research on medical students’ willingness to work on the frontline has focused mainly on the COVID-19 pandemic. Studies among medical students have reported willingness rates to engage in frontline work during the pandemic ranging from 48.7% to 81% [15–19]. However, no studies have examined medical students’ willingness to undertake frontline duties during mpox outbreaks. Data on medical students’ knowledge, attitudes, and practices (KAP) toward mpox and the association of KAP with willingness to work on the mpox frontline remain limited.

Accordingly, we aimed to investigate Chinese medical students’ willingness to engage in frontline work during a potential mpox outbreak and to explore associated factors in a cross-sectional study. We address a gap in evidence on the response of the reserve medical workforce to mpox, enrich the evidence base for cross-disease and cross-regional comparative analyses, and provide empirical insights for medical education institutions seeking to optimise emergency response training, strengthen professional value formation, and develop targeted mobilisation strategies. We also estimate the potential contribution of the medical student reserve workforce, providing a reference for improving the resilience and responsiveness of China’s public health emergency system.

## METHODS

### Study design

We conducted a nationwide cross-sectional survey from November 2023 to March 2024. We used snowball sampling for participant recruitment. As a non-probability sampling method, it is an important methodological limitation, as it carries an inherent risk of selection bias and may limit the representativeness of the sample. We reported this study in accordance with the STROBE guidelines [20].

### Participants and survey procedures

The survey included a total of 4,320 full-time medical students across mainland China. We obtained 4,098 valid questionnaires, yielding a valid response rate of 94.86%.

We conducted an anonymous online survey using Wenjuanxing, a professional online survey platform in China. Researchers generated a QR code for the questionnaire through this platform and sent it to counsellors. The counsellors disseminated the QR code to potential participants through QQ and WeChat. Participants were also encouraged to assist recruitment by sharing the questionnaire QR code with other eligible students. All respondents voluntarily used digital devices to scan the code and complete the online questionnaire. To ensure data integrity, every item was set as a required field, and only one submission was allowed per IP address to avoid duplicate responses. In addition, Wenjuanxing automatically recorded response time; questionnaires completed in fewer than 180 seconds were regarded as invalid and excluded from analysis.

### Survey instrument

The questionnaire was developed according to the Technical Guidelines for Mpox Prevention and Control (2022) [11]. Before distribution, independent public health experts reviewed the questionnaire to assess content validity and adequacy. We then conducted a pilot test to evaluate item comprehensibility. Internal consistency was assessed using Cronbach’s α, which yielded a value of 0.916, indicating high internal reliability. The questionnaire comprised six parts and 78 items: basic information (16 items), knowledge (23 items), attitude (11 items), practice (five items), vaccination-related behaviours (22 items), and medical students’ willingness to work on the frontline during an mpox outbreak (one item). In the knowledge section, single-choice items were scored as two points for correct answers and 0 points for incorrect answers, whereas multiple-choice items awarded one point for each accurately selected option. The attitude, practice, vaccination-related behaviour, and willingness sections were assessed using a five-point Likert scale. For willingness to work on the frontline, scores of 1–2 indicated refusal, a score of 3 denoted hesitancy, and scores of 4–5 represented acceptance of frontline work during an mpox outbreak. The total questionnaire score was 250 points, with maximum scores of 60, 55, 25, and 110 points for the knowledge, attitude, practice, and vaccination-related behaviour sections, respectively. Higher scores in each section denoted greater mpox-related knowledge, more positive attitudes, better preventive and protective practices, and more favourable vaccination-related behaviours.

### Statistical analysis

We summarised categorical variables as frequencies and percentages and performed between-group comparisons using the χ^2^ test or Fisher exact test. We used the Kolmogorov-Smirnov test to examine the normality of continuous variables. We presented normally distributed continuous variables as means and standard deviations (SDs), and analysed intergroup differences using the *t*-test or one-way analysis of variance. We expressed non-normally distributed continuous variables as medians and interquartile ranges (IQRs), and made comparisons using the Kruskal-Wallis *H* test. We performed multinomial logistic regression to identify factors associated with willingness to work on the frontline during an mpox outbreak. We reported associations as odds ratios (ORs) and corresponding 95% confidence intervals (CIs). We used willingness to work on the frontline during an mpox outbreak as the dependent variable (Acceptance = 1, Hesitancy = 2, Refusal = 3). In the multinomial logistic regression model, the acceptance group served as the reference category for the dependent variable. We entered variables with *P* < 0.10 in univariable analyses as independent variables [21–24]. We used a stepwise selection procedure to retain variables in the final model. We considered *P* < 0.05 (two-sided) statistically significant.

We performed all statistical analyses using SPSS, version 24.0 (International Business Machines Corporation, Armonk, New York, USA) software.

## RESULTS

### Demographic characteristics and willingness of medical students to work on the mpox frontline

We included 4098 medical students in mainland China, of whom 1,739 (42.44%) were male, and 2,359 (57.56%) were female. Among all participants, 3,158 (77.06%) were willing to participate in frontline work during an mpox outbreak, 761 (18.57%) reported hesitancy, and 179 (4.37%) explicitly refused.

Medical students’ willingness to work on the mpox frontline was significantly associated with grade, only-child status, student leader status, reason for choosing medicine, self-rated health status, chronic disease, organic disease, and family members or friends who had experienced COVID-19. Compared with their counterparts, students in lower grades, non-only children, student leaders, students who chose medicine based on their own preference, students with better self-rated health, students without chronic or organic disease, and students whose family members or friends had experienced COVID-19 showed significantly greater willingness to work on the mpox frontline (**Table 1**).

**Table 1:** Demographic characteristics of medical students and their willingness to work on the mpox frontline*

		Willingness to work				
	**All participants**	**Acceptance**	**Hesitancy**	**Rejection**	***χ* ^2^**	***P*-value**
**Total**	4,098 (100)	3,158 (77.06)	761 (18.57)	179 (4.37)		
**Gender**					3.8522	0.1457
Male	1,739 (42.44)	1,314 (75.56)	344 (19.78)	81 (4.66)		
Female	2,359 (57.56)	1,844 (78.17)	417 (17.68)	98 (4.15)		
**Grade**					35.1085	<0.0001
First	1,731 (42.24)	1,389 (80.24)	281 (16.23)	61 (3.52)		
Second	911 (22.23)	721 (79.14)	154 (16.90)	36 (3.95)		
Third	880 (21.47)	641 (72.84)	192 (21.82)	47 (5.34)		
Fourth	314 (7.66)	222 (70.70)	73 (23.25)	19 (6.05)		
Fifth	262 (6.39)	185 (70.61)	61 (23.28)	16 (6.11)		
**Major**					5.1032	0.0780
Clinical medicine	3,009 (73.43)	2,293 (76.20)	583 (19.38)	133 (4.42)		
Others	1,089 (26.57)	865 (79.43)	178 (16.35)	46 (4.22)		
**Region**					5.2678	0.0718
Urban areas	2,365 (57.71)	1,792 (75.77)	464 (19.62)	109 (4.61)		
Rural areas	1,733 (42.29)	1,366 (78.82)	297 (17.14)	70 (4.04)		
**Only child**					12.1347	0.0023
Yes	1,727 (42.14)	1,285 (74.41)	355 (20.55)	87 (5.04)		
No	2,371 (57.86)	1,873 (79.00)	406 (17.12)	92 (3.88)		
**Poor student identified by the school**					0.0798	0.9609
Yes	1,002 (24.45)	769 (76.75)	189 (18.86)	44 (4.39)		
No	3,096 (75.55)	2,389 (77.16)	572 (18.48)	135 (4.36)		
**Student leader**					6.0931	0.0475
Yes	1,043 (25.45)	832 (79.77)	168 (16.11)	43 (4.12)		
No	3,055 (74.55)	2,326 (76.14)	593 (19.41)	136 (4.45)		
**The reason for choosing medicine**					33.8316	<0.0001
Own desire	3,153 (76.94)	2,495 (79.13)	537 (17.03)	121 (3.84)		
Suggested by others	945 (23.06)	663 (70.16)	224 (23.70)	58 (6.14)		
**Do your parents or relatives have medical workers?**					4.7122	0.0948
Yes	1,540 (37.58)	1,213 (78.77)	260 (16.88)	67 (4.35)		
No	2,558(62.42)	1,945 (76.04)	501 (19.59)	112 (4.38)		
**Do your parents or relatives engage in frontline work during the COVID-19 outbreak?**					4.8723	0.0875
Yes	822 (20.06)	656 (79.80)	131 (15.94)	35 (4.26)		
No	3,276 (79.94)	2,502 (76.37)	630 (19.23)	144 (4.40)		
**Do you have internship experience?**					2.1930	0.3340
Yes	1,254 (30.60)	948 (75.60)	248 (19.78)	58 (4.62)		
No	2,844 (69.40)	2,210 (77.71)	513 (18.04)	121 (4.25)		
**Self-health status**					50.9192	<0.0001
Very poor	66 (1.61)	41 (62.12)	13 (19.70)	12 (18.18)		
Poor	209 (5.10)	149 (71.29)	47 (22.49)	13 (6.22)		
Average	1,693 (41.31)	1,268 (74.90)	351 (20.73)	74 (4.37)		
Good	1,448 (35.33)	1,145 (79.07)	247 (17.06)	56 (3.87)		
Very good	682 (16.64)	555 (81.38)	103 (15.10)	24 (3.52)		
**Chronic disease**					9.2977	0.0096
Yes	329 (8.03)	249 (75.68)	55 (16.72)	25 (7.60)		
No	3,769 (91.97)	2,909 (77.18)	706 (18.73)	154 (4.09)		
**Organic disease**					13.2932	0.0013
Yes	143 (3.49)	103 (72.03)	25 (17.48)	15 (10.49)		
No	3,955 (96.51)	3,055 (77.24)	736 (18.61)	164 (4.15)		
**Have you, your relatives, or friends ever had COVID-19?**					24.7153	<0.0001
Yes	3,566 (87.02)	2,793 (78.32)	626 (17.55)	147 (4.12)		
No	532 (12.98)	365 (68.61)	135 (25.38)	32 (6.02)		
**Were you placed under centralised quarantine due to the COVID-19 pandemic?**					2.0420	0.3602
Yes	1,244 (30.36)	943 (75.80)	240 (19.29)	61 (4.90)		
No	2,854 (69.64)	2,215 (77.61)	521 (18.26)	118 (4.13)		

*Presented as n (%).

### Mpox-related KAP status among medical students and its association with willingness to work on the mpox frontline

The median scores for mpox knowledge, attitude, practice, and vaccination-related behaviours among medical students were 43 (IQR = 33–50), 33 (IQR = 32–36), 20 (IQR = 19–24), and 73 (IQR = 68–79), respectively. The median scores for mpox knowledge (H = 161.9005; *P* < 0.0001), practice (H = 1452.1984; *P* < 0.0001), and vaccination-related behaviours (H = 615.5106; *P* < 0.0001) were significantly associated with medical students’ willingness to work on the mpox frontline, whereas the mpox attitude score (H = 2.2951; *P* = 0.3174) showed no significant association (**Figure 1**, **Table 2**).

**Figure 1:**
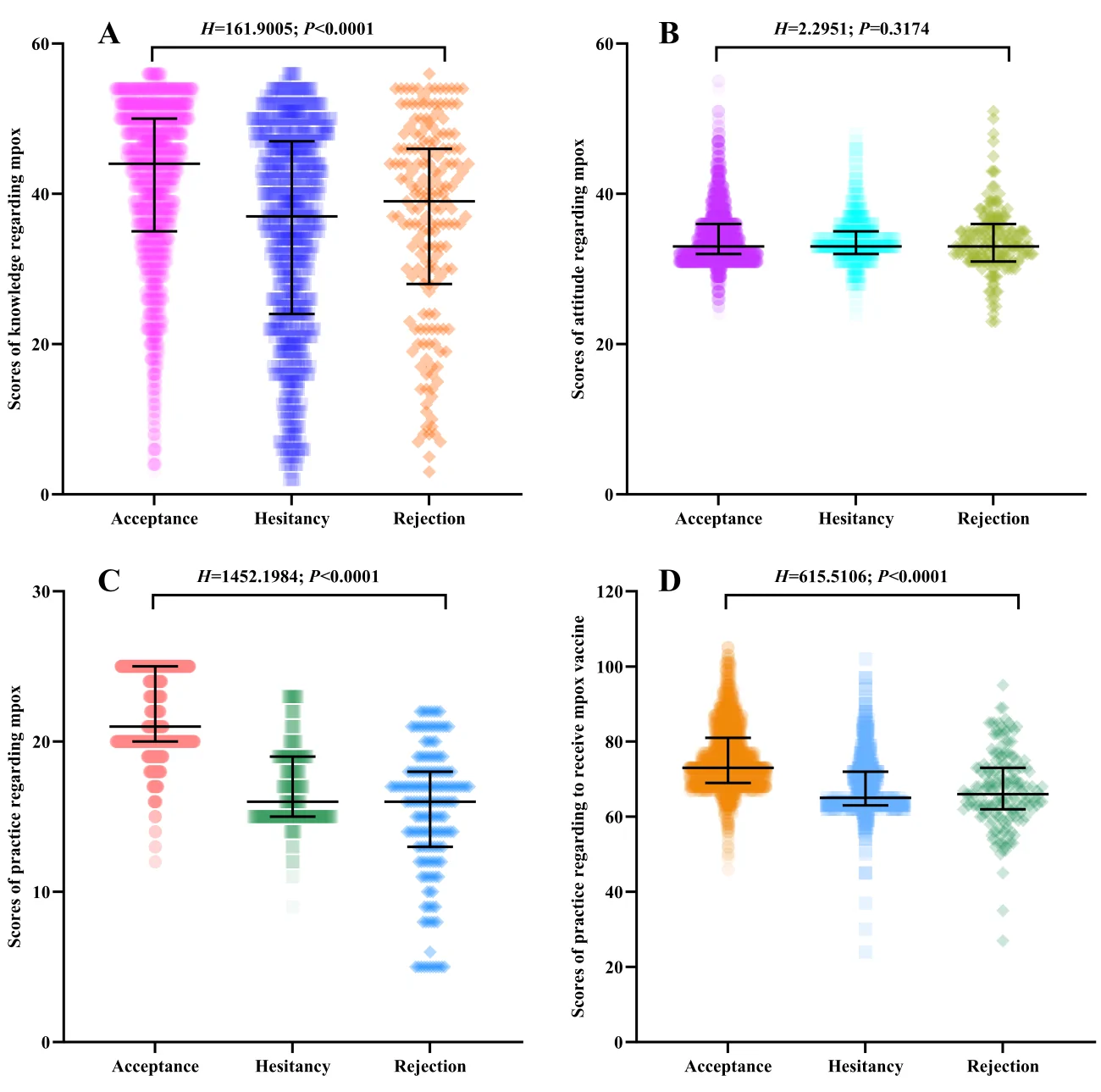
Willingness to work on the mpox frontline by knowledge, attitudes and practices status and mpox vaccination status among medical students. **Panel A.** Knowledge scores regarding mpox. **Panel B.** Attitude scores regarding mpox. **Panel C.** Practice scores regarding mpox. **Panel D.** Practice scores regarding receiving the mpox vaccine.

**Table 2:** Knowledge, attitudes and practices status regarding mpox among medical students and its association with their willingness to work on the mpox frontline*

		Willingness to work				
	**All participants**	**Acceptance**	**Hesitancy**	**Rejection**	**H**	***P*-value**
**Knowledge**	43 (33–50)	44 (35–50)	37 (24–47)	39 (28–46)	161.9005	<0.0001
**Attitude**	33 (32–36)	33 (32–36)	33 (32–35)	33 (31–36)	2.2951	0.3174
**Practice**	20 (19–24)	21 (20–25)	16 (15–19)	16 (13–18)	1452.1984	<0.0001
**Practice to receive mpox vaccine**	73 (68–79)	73 (69–81)	65 (63–72)	66 (62–73)	615.5106	<0.0001

*Presented as medians (interquartile ranges).

### Multinomial logistic regression for willingness to work on the mpox frontline

Compared with the acceptance group, grade, major, region, only-child status, reason for choosing medicine, whether parents or relatives had engaged in frontline work during the COVID-19 outbreak, mpox knowledge status, and vaccination-related behaviour score were significantly associated with hesitancy to work on the mpox frontline. Compared with their reference groups, students in higher grades, clinical medicine students, urban students, only children, students who chose medicine at others’ suggestion, and students whose parents or relatives had no frontline work experience during the COVID-19 outbreak had higher odds of hesitancy. Higher mpox knowledge scores (OR = 0.973; 95% CI = 0.966–0.981, *P* < 0.0001) and higher vaccination-related behaviour scores (OR = 0.894; 95% CI = 0.882–0.907, *P* < 0.0001) were associated with lower odds of hesitancy (**Figure 2**, **Table 3**).

**Figure 2:**
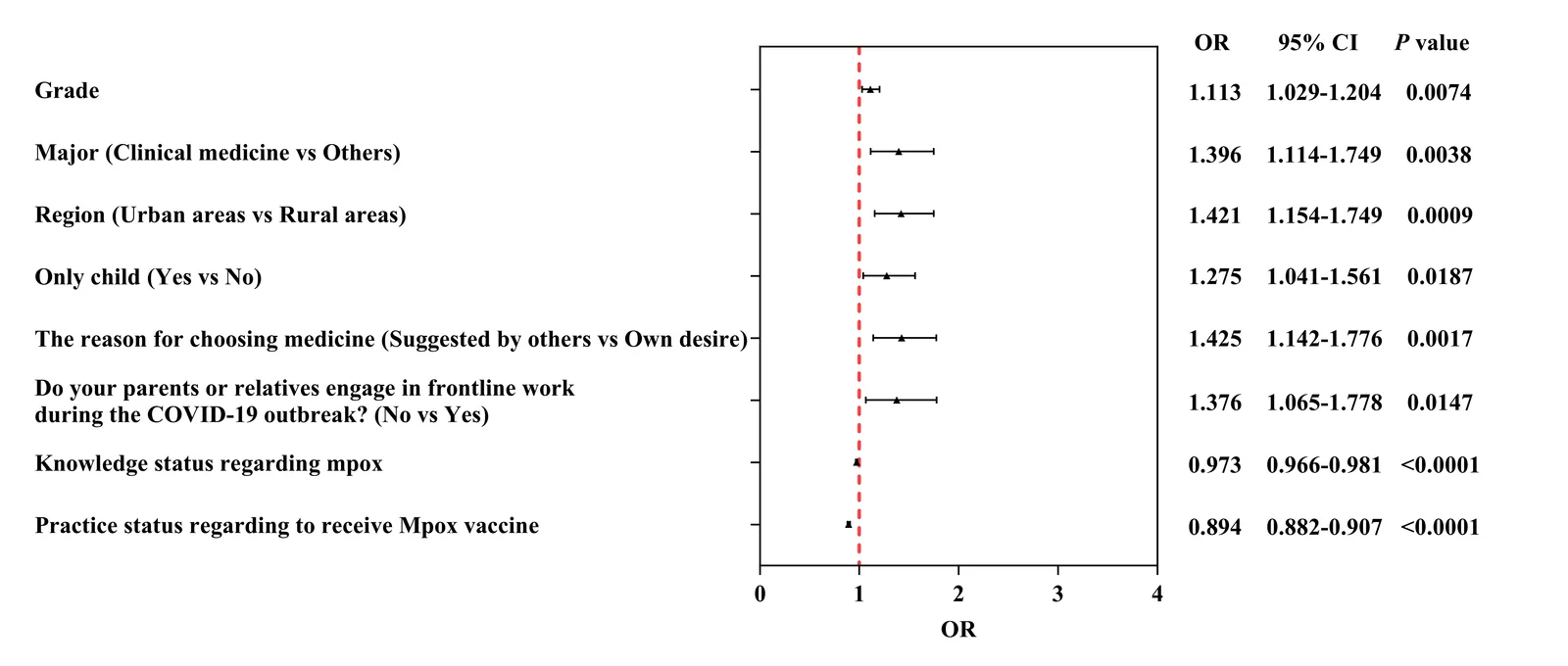
Multinomial logistic regression analysis of the determinants significantly associated with hesitancy to work on the mpox frontline.

**Table 3:** Factors correlated with the willingness to work on the mpox frontline*

	*β*	S_x̄_	Wald*χ*^2^	*P*-value	OR (95% CI)
**Hesitancy**					
Grade	0.1072	0.0400	7.1791	0.0074	1.113 (1.029–1.204)
**Major (others)**					
Clinical Medicine	0.3336	0.1152	8.3884	0.0038	1.396 (1.114–1.749)
**Region (rural areas)**					
Urban areas	0.3514	0.1061	10.9692	0.0009	1.421 (1.154–1.749)
**Only child (no)**					
Yes	0.2428	0.1032	5.5315	0.0187	1.275 (1.041–1.561)
**The reason for choosing medicine (own desire)**					
Suggested by others	0.3538	0.1127	9.8656	0.0017	1.425 (1.142–1.776)
**Do your parents or relatives engage in frontline work during the COVID-19 outbreak? (Yes)**					
No	0.3192	0.1308	5.9580	0.0147	1.376 (1.065–1.778)
**Knowledge status regarding mpox**	−0.0271	0.0040	46.9228	<0.0001	0.973 (0.966–0.981)
Practice status regarding receiving the mpox vaccine	−0.1115	0.0073	235.6546	<0.0001	0.894 (0.882–0.907)
**Rejection**					
Grade	0.1392	0.0705	3.8942	0.0485	1.149 (1.001–1.320)
**The reason for choosing medicine (own desire)**					
Suggested by others	0.4916	0.1974	6.2041	0.0127	1.635 (1.110–2.407)
**Organic disease (No)**					
Yes	0.9256	0.3386	7.4711	0.0063	2.523 (1.299–4.901)
Practice status regarding receiving the mpox vaccine	−0.1247	0.0123	102.8110	<0.0001	0.883 (0.862–0.904)

CI – confidence interval, OR – odds ratio

*The brackets represent the reference group. The willingness to work on the mpox frontline was used as the dependent variable, which included three categories: acceptance, hesitancy, and rejection. In the multinomial logistic regression model, the status of the acceptance group served as the reference category for the dependent variable.

Compared with the acceptance group, grade, reason for choosing medicine, organic disease, and vaccination-related behaviour score were significantly associated with refusal. Compared with their reference groups, students in higher grades, students who chose medicine at others’ suggestion, and students with organic disease had higher odds of refusal. Higher vaccination-related behaviour scores (OR = 0.883; 95% CI = 0.862–0.904, *P* < 0.0001) were associated with lower odds of refusal (**Figure 3**, **Table 3**).

**Figure 3:**
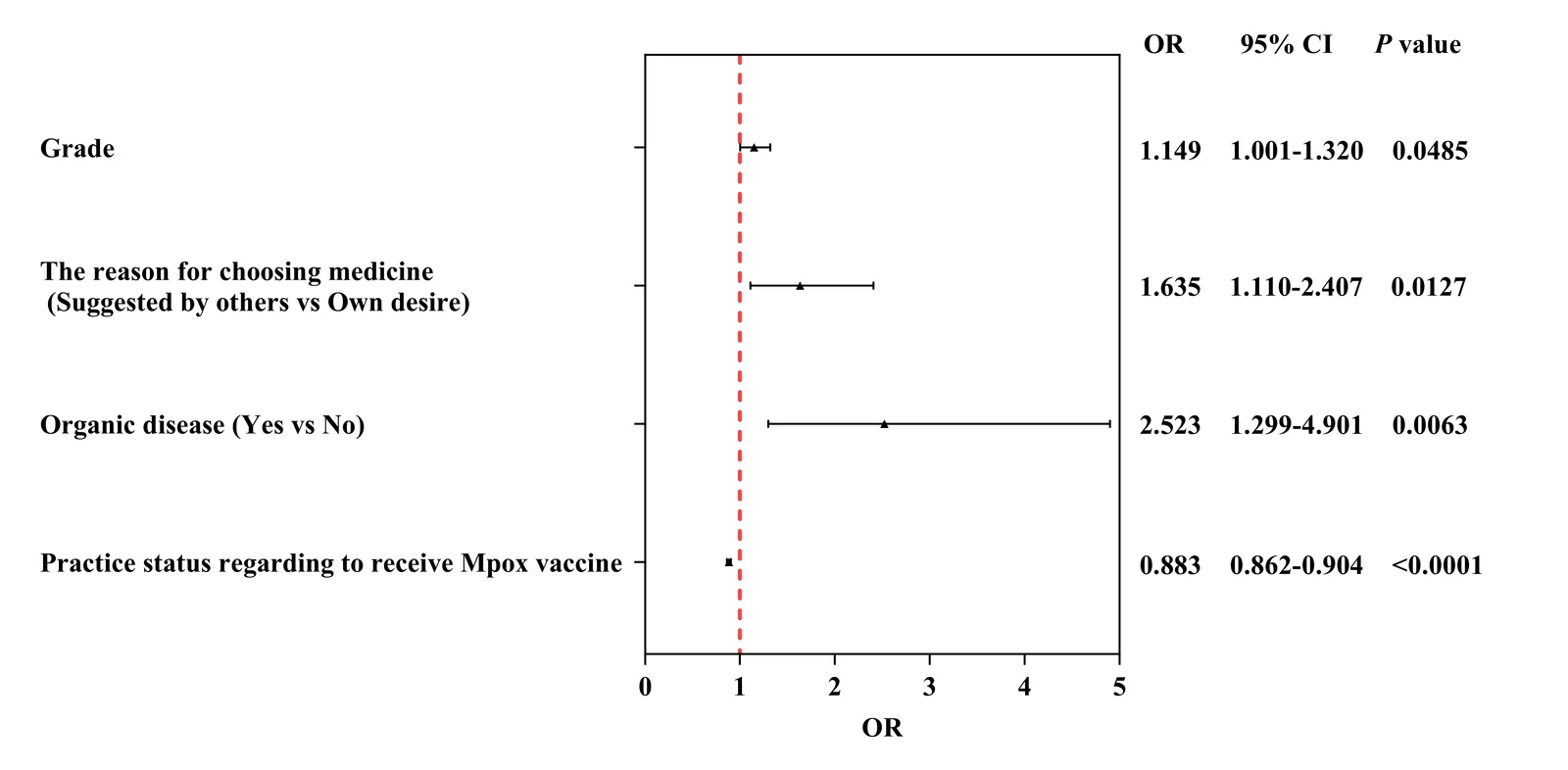
Multinomial logistic regression analysis of the determinants significantly associated with rejection of working on the mpox frontline.

## DISCUSSION

This nationwide cross-sectional study enrolled 4098 medical students in mainland China to examine their willingness to support frontline responses during potential mpox outbreaks and to identify demographic, health-related, behavioural, and mpox-related KAP factors associated with this willingness. To our knowledge, this is the first large-scale study to focus on medical students’ willingness to participate in frontline mpox response. Our study provides empirical evidence to strengthen public health emergency preparedness and develop targeted strategies for mobilising medical reserve personnel. Furthermore, we address a key research gap in mpox preparedness and provide an evidence base for supporting and deploying the medical student workforce in response to mpox and future emerging infectious diseases.

In our study, 77.06% of medical students were willing to undertake frontline duties during mpox outbreaks, 18.57% were hesitant, and only 4.37% refused. This willingness rate is generally consistent with or higher than findings from COVID-19 studies among medical students in Asia. Published willingness levels during the pandemic were 48.7% in Indonesia, 66.3% in South Korea, 72.5% in Vietnam, and 81% in Singapore [15–18]; the Singaporean estimate was derived under a scenario assuming a case fatality rate of less than 10%. Compared with these international data, the willingness level in our study was close to that in Singapore and notably higher than those in Indonesia and Vietnam. However, the voluntary participation and snowball sampling approach may introduce positive selection bias, whereby students with stronger engagement and greater attention to emerging infectious disease response are more likely to complete the survey. Consequently, the 77.06% willingness estimate may represent an overestimation among medical students in mainland China. Even accounting for this potential bias, the relatively high willingness observed in this study may still be broadly attributable to three contextual factors: China’s well-established public health emergency system [9], enhanced professional responsibility shaped by medical ethics and social mission education [25], and the low epidemic intensity of mpox in China [8], which may jointly reduce fear-driven avoidance.

Notably, some variables showed non-significant crude associations in χ^2^ analyses but emerged as significant independent correlates in the adjusted multinomial logistic regression model. This discrepancy is common in observational research, as confounding factors may mask underlying associations in unadjusted analyses. Meanwhile, variables including sex showed no statistically significant crude associations in χ^2^ tests and did not emerge as independent correlates in the adjusted model, suggesting limited independent association with frontline work willingness in this study sample. Therefore, the adjusted findings from the multinomial logistic regression should be given greater weight when interpreting the independent determinants of willingness, because they account for confounding. The strength of associations between participant characteristics and frontline work willingness varied notably across variables, highlighting the need to interpret findings beyond statistical significance alone. The weak association between higher grade and increased hesitancy should be interpreted with caution, as its limited practical relevance may be partially driven by the large sample size. In comparison, clinical medicine majors showed a stronger association with hesitancy, making it a more meaningful correlate of willingness differences. These two findings may share a common underlying mechanism linked to clinical training and risk perception. Students in higher grades and clinical medicine majors typically receive more systematic clinical training and professional knowledge, which fosters a more comprehensive understanding of occupational risks and infection control challenges in infectious disease outbreaks. Accordingly, greater professional exposure may lead to more prudent assessments of the risks and responsibilities involved in mpox frontline work [26,27]. For frontline work refusal, the association with higher grade also showed a weak effect size, and thus warrants cautious interpretation. This pattern aligns with findings from prior public health emergencies, where greater professional knowledge and risk awareness have been linked to more prudent attitudes toward frontline participation [28,29].

Consistent with the perceived barriers dimension of the Health Belief Model (HBM) [30], both urban residence and only-child status were linked to increased frontline work hesitancy in this study, though the association for only-child status was relatively weak and requires cautious interpretation. Within the HBM, perceived barriers refer to the subjective obstacles individuals perceive when engaging in a health behaviour [31,32]. Students from urban areas may have access to more abundant and diverse epidemic-related information, which may increase perceived disease severity and thus increase hesitancy in decision-making. Although the effect size is modest, the association for only-child students can still be interpreted from the perspective of perceived barriers and Chinese family culture. Only children in China typically bear greater elder care responsibilities within the family; their concerns about personal health risks from frontline work, alongside worries about impacts on family functioning, may create higher psychological barriers to participation. This pattern appears consistent with our previous findings on mpox vaccination willingness, in which only-child status was also linked to greater hesitancy, suggesting a relatively consistent association across different mpox-related health behaviours [33].

The associations between choosing medicine out of intrinsic motivation and greater frontline willingness, and between absence of family COVID-19 frontline experience and higher hesitancy, can be interpreted through intrinsic motivation and observational learning. First, students who chose the medical profession based on intrinsic motivation made career choices rooted in autonomous value judgments. Their professional identity in medicine and ethical responsibility have become core components of their self-concept [34]. In our study, medical students driven by intrinsic motivation were more likely to view frontline work during public health crises as an opportunity to practice professional values rather than merely as a risk event, thereby demonstrating higher acceptance of such roles. Second, frontline experience among parents or relatives during the COVID-19 pandemic may influence medical students’ work willingness through value transmission and risk information. Family members with pandemic response experience may convey recognition of the social value of public health work and the ethical responsibility to step forward during crises. This family-level value transmission may subtly shape medical students’ professional attitudes. This information may reduce medical students’ fear of the unknown regarding frontline work and thereby decrease hesitancy.

For the association between organic disease and frontline work refusal, the effect estimate was relatively strong but accompanied by a wide confidence interval, indicating notable uncertainty in the magnitude of the association. This finding should therefore be interpreted cautiously and warrants confirmation in future studies with a larger number of participants with underlying diseases. From the perspective of perceived severity within the HBM, students with organic disease may perceive themselves as being more vulnerable to adverse health consequences, and could adopt more cautious attitudes toward frontline work. Our previous study also found that organic disease was associated with mpox vaccine refusal [33].

Mpox knowledge and practice scores demonstrated significant associations with such willingness. This finding diverges from the classical assumptions of the KAP model, which conventionally posits that knowledge shapes behaviour indirectly through attitude, forming a progressive knowledge–attitude–practice pathway. As an emerging infectious disease that gained global prominence only after 2022, mpox has not yet fostered a stable, systematic attitudinal framework among the general public, including medical students. When responding to mpox-related attitude items, medical students may express transient affective reactions to ambiguous contexts rather than deep judgments consolidated through cognitive integration. By contrast, knowledge and practice domains directly reflect an individual’s cognitive preparedness and behavioural readiness toward mpox. These domains therefore have more direct and actionable links with frontline work willingness [35].

Higher mpox knowledge scores were associated with lower hesitancy. This finding is consistent with a prior cross-sectional study of 579 medical students [36], which identified mastery of disease-related knowledge and previous disaster training as key determinants of willingness to work during emerging infectious disease outbreaks. Students with the highest knowledge levels also exhibited the strongest willingness to respond [36]. Therefore, adequate knowledge of mpox may enable individuals to assess transmission risks accurately and implement effective protective strategies, thereby strengthening willingness to engage in frontline activities. Moreover, improved knowledge may heighten sensitivity to public health emergency demands, encouraging medical students to translate professional competence into practical action during outbreaks.

Similarly, higher mpox vaccination-related behaviour scores were strongly associated with lower odds of hesitancy and refusal. This finding suggests that vaccination behaviour represents not only a concrete measure for infectious disease control but also an observable behavioural indicator of individual acceptance of public health interventions. Han *et al.* reported that prior influenza or COVID-19 vaccination was associated with higher mpox vaccine acceptance, supporting this predictive relationship [37].

Importantly, the deployment of medical students as an emergency reserve workforce must be underpinned by robust ethical governance, given that willingness alone does not guarantee clinical competence or appropriate deployment conditions. First, the lower willingness observed among senior clinical students may partially stem from their richer clinical exposure and more comprehensive awareness of occupational risks in outbreak settings, which highlights that full, transparent risk disclosure is a foundational prerequisite for any recruitment. Second, patient safety must remain the top priority: medical students should only undertake auxiliary and supportive tasks under the direct supervision of fully licensed healthcare providers, rather than independent clinical duties, to ensure appropriate and safe patient care. Third, the principle of strictly voluntary participation must be enforced, with explicit safeguards to eliminate implicit pressure linked to academic assessment, performance evaluation or graduation requirements, and a penalty-free withdrawal mechanism should be available throughout the deployment period. Fourth, clear supervision hierarchies and legal liability frameworks are essential: a dedicated preceptor accountability system should be implemented to clarify scope of authority and responsibility, and supporting legal protections and medical liability insurance should be provided to address risks associated with potential clinical errors. Finally, students are entitled to reasonable compensation and occupational security, including appropriate work subsidies, adequate personal protective equipment, work injury insurance, and guaranteed medical care and support in the event of occupational infection.

### Strengths and limitations

Notably, this study is the first nationwide, large-scale investigation of medical students’ frontline willingness toward mpox, and the inclusion of 4098 participants provides a substantial empirical sample. We integrated demographic, health-related, mpox-related KAP, and mpox vaccine-related behaviour factors, providing preliminary empirical evidence and practical references to inform the development of a public health emergency reserve workforce and advance emergency preparedness. Several limitations should be acknowledged. First, snowball sampling with voluntary participation may introduce positive selection bias, so representativeness cannot be fully assured. Second, no formal a priori sample size calculation was performed in this study. Given the non-probability nature of snowball sampling and the absence of a predefined sampling frame, traditional probability-based sample size estimation methods are not applicable, representing a methodological limitation. Third, self-reported data may be subject to social desirability bias, potentially overestimating willingness. Fourth, the cross-sectional design precludes causal inference. Fifth, the sample was restricted to medical students in mainland China, limiting generalisability to countries with different educational systems, outbreak experiences, and public health responses. Additionally, this study did not assess actual clinical competence, detailed risk perceptions, or attitudes toward the aforementioned ethical safeguards, all of which merit further exploration.

## CONCLUSIONS

This is the first large-scale national survey in China to assess medical students’ willingness to work on the mpox frontline and its associated factors. Overall, 77.06% were willing, whereas 22.94% were hesitant or refused. Frontline response willingness was independently associated with grade, only-child status, professional motivation, health status, mpox-related knowledge, practice, and vaccination-related behaviours. Medical schools should integrate emerging infectious disease training into core curricula, provide psychological support for higher-risk groups, and develop tailored mobilisation strategies to strengthen the reserve medical workforce for future public health emergencies.

## Additional material


Online Supplementary Document

## Data Availability

**Data availability:** The data sets used and/or analysed during the current study are available from the corresponding author at superyuehui@163.com upon reasonable request.
