# Supplementary material for: Willingness of Chinese medical students to work on the frontline during a potential mpox outbreak: a nationwide cross-sectional study
Source: J Glob Health. 2026 Nov 6;16:04327. doi: 10.7189/jogh.16.04327 (PMC13741167; doi:10.7189/jogh.16.04327)
Supplement: Supplementary file 1 [file jogh-16-04327-s001.pdf]

Supplement to: Chen J, Ge J, Han Y, Xie Z, Li H, Zhao Y, Jia Y. Willingness of Chinese medical students to work on the frontline during a potential mpox outbreak: a nationwide cross-sectional study. J Glob Health. 2026;16:04327.

Interviewee's informed consent

Hello, dear classmates!

We are researchers from Qiqihar Medical University. You are welcome to participate in this survey. Your participation in this research is entirely voluntary. Therefore, you may opt to decline or withdraw at any time without any penalty. This survey is conducted in an anonymous form. We will keep your information strictly confidential. Please read the following informed consent carefully.

If you have any questions about this study or this consent form, please contact the principal researcher (refer to Article 10). If you decide to participate in this research, then you must sign this consent form and select 'Agree' at the end.

1. The name of the research project:

Knowledge–attitude–practice status of college students during a potential mpox outbreak: a cross-sectional study.

2. The main researchers:

Jiaxin Chen and Yuehui Jia

3. The research purpose of the project:

This study aimed to investigate the knowledge–attitude–practice (KAP) of Chinese college students during a potential mpox outbreak.

4. The research content of the project:

Basic survey information and questionnaire for knowledge–attitude–practice regarding mpox.

5. What do you need to do in this survey?

If you agree to participate in this survey, then you only need to answer the survey questions herein according to your own experiences or actual situations.

6. What are the possible risks or discomforts?

This survey has no foreseeable risks.

7. What possible benefits can the participant obtain from this survey?

Participating in this survey can increase your knowledge regarding mpox and promote healthy behavior. This survey will benefit others in the future, because it will be provide a basis for the government to adopt targeted health education and precise prevention and control strategies, along with data support for the psychological intervention of college students during the pandemic.

8. You can opt to participate in or withdraw from this survey at any time voluntarily.

Your participation in the research is entirely voluntary. For this survey, you have the right to withdraw at any time without any penalty.

9. How will your personal privacy be protected in this survey?

This questionnaire survey is anonymous, and your survey records will be saved in a special way (in the form of encrypted files) to ensure that only the researchers of this project can access it. When necessary, only members of the ethics committee and government management can access your records.

The results of this survey may be published in scientific journals. Given that the data are collected anonymously, please rest assured that your name or other personally identifiable information will not appear.

10. Who can you contact for questions and clarifications?

If you have any questions or confusion about participating in the survey or have any queries about your rights as a survey participant during the research process, you may ask the person in charge of the research at any time. The principal researcher email: superyuehui@163.com.

I have read the introduction about this research. I know the risks and benefits that may arise from participating in this research. I understand that my participation in this research is voluntary, and I confirm that I have sufficient time to consider it. I also understand that I can consult the researchers for other information at any time, and I can withdraw from this research at any time without discrimination or retaliation.

Do you agree in participating this survey?

○ Agree（Proceed to the first question of the questionnaire）

○ Disagree（Proceed to the end of the questionnaire）

Questionnaire on Medical Students' Knowledge, Attitudes, Behaviors and Vaccination Willingness Regarding Mpox

Dear students,

Hello! In June 2023, local cases of mpox began to emerge in China. This is a questionnaire about college students' knowledge, attitudes, behaviors and psychological status regarding mpox. Please answer the following questions according to your true situation. Your real thoughts will be of great help to us. This survey is completely anonymous, and your information will not be disclosed to the outside world. The survey results will only be used for scientific research. Please feel free to fill it out. Thank you sincerely for your enthusiastic participation!

Basic information

1. Gender:
   A. Male
   B. Female

2. Where is your home located?
   A. Urban area
   B. Rural area

3. Are you an only child in your family?
   A. Yes
   B. No

4. Grade:
   A. First
   B. Second
   C. Third
   D. Fourth
   E. Fifth

5. Major:
   A. Clinical medicine
   B. Stomatology
   C. Anesthesiology

D. Preventive medicine

E. Nursing medicine

F. Medical imaging

G. Medical Examination

H. Psychiatry

I. Rehabilitation

J. Other major

6. Are you a poor student identified by the school?

A. Yes

B. No

7. Are you a student leader?

A. Yes

B. No

8. The reason for choosing medicine.

A. Own desire

B. Suggested by others

9. Do your parents or relatives have medical workers?

A. Yes

B. No

10. During the COVID-19 pandemic, have any of your parents or relatives had experience working on the frontline of the fight against the virus?

A. Yes

B. No

11. Do you have internship experience?

A. Yes

B. No

12. How would you describe your current physical health status?

A. very poor

B. Poor

C. Fair

D. Good

F. very good

13. Have you ever been diagnosed with a chronic disease?

A. Yes

B. No

14. Are you an individual with an allergic constitution?

A. Yes

B. No

15. Do you currently have any organic diseases (diagnosed through medical examinations in a hospital)? (Organic lesions refer to permanent damage to one or more of the body's organ systems caused by one or more factors, such as inflammation, ulcers, tumors, etc. Such organic lesions cannot be completely cured, and only medications can be used to relieve symptoms.)

A. Yes

B. No

16. Have you yourself, or any of your relatives or friends, ever been diagnosed with COVID-19?

A. Yes

B. No

Survey on Awareness of Mpox Virus

17.mpox is an infectious disease.

A. Yes

B. No

C. Don't know

18.mpox is a viral zoonotic disease caused by infection with the mpox virus.

A. Yes

B. No

C. Don't know

19.mpox can be transmitted from person to person.

A. Yes

B. No

C. Don't know

20.The mpox virus is resistant to dryness and low temperatures, and can survive for several months in soil, scabs, and clothing/bedding.

A. Yes

B. No

C. Don't know

21.The mpox virus is sensitive to heat and can be inactivated by heating at 56°C for 30 minutes or at 60°C for 10 minutes.

A. Yes

B. No

C. Don't know

22.Historically, mpox has primarily occurred in Central Africa and West Africa.

A. Yes

B. No

C. Don't know

23.The case fatality rate (CFR) of mpox ranges from 1% to 10%.

A. Yes

B. No

C. Don't know

24. The main clinical manifestations of mpox include (multiple choices):

A. Fever

B. Rash

C. Lymphadenopathy

D. Dry Cough

25. The symptoms of mpox and smallpox are similar:

A. Yes

B. No

C. Don't know

26.The main hosts of the mpox virus include (multiple choices):

A. African rodents (e.g., African squirrels, tree squirrels, Gambian pouched rats, dormice, etc.)

B. Primates (e.g., various species of monkeys and apes)

C. Pets such as cats and dogs

D. Poultry such as chickens, ducks and geese

E. Livestock such as pigs, cattle, sheep and horses

27. Animals infected with the mpox virus and individuals infected with the mpox virus are the main sources of infection.

A. Yes

B. No

C. Don't know

28. Populations who have not been vaccinated against smallpox are generally susceptible to the mpox virus.

A. Yes

B. No

C. Don't know

29. The mpox virus invades the human body through mucous membranes and broken skin.

A. Yes

B. No

C. Don't know

30. Transmission Routes of the mpox Virus Include: (Multiple Choices)

A. Contact with respiratory secretions, lesion exudates, blood, and other body fluids of infected animals.

B. Infection through bites or scratches from infected animals.

C. Among humans, transmission mainly occurs through close contact.

D. Among humans, transmission can occur through respiratory droplets during prolonged close-range contact.

E. Infection may also occur through contact with items contaminated by the mpox virus.

F. The mpox virus can also be transmitted from pregnant women to fetuses via the placenta.

31. The incubation period of mpox ranges from 5 to 21 days, and it is mostly 6 to 13 days.

A. Yes

B. No

C. Don't know

32. mpox is a self-limiting disease. (A self-limiting disease refers to a disease that can stop automatically after developing to a certain extent, gradually recover and heal without the need for special treatment. It only requires symptomatic treatment or no treatment at all, and can be cured by the body's own immunity. Examples include viral infections, autoimmune diseases, etc.)

A. Yes

B. No

C. Don't know

33.Since May 2022, the mpox outbreaks that have occurred in multiple countries around the world have mainly spread through sexual contact among men who have sex with men (MSM), and most of the cases have been young and middle-aged men.

A. Yes

B. No

C. Don't know

34.Regarding the specific measures to prevent mpox infection, which of the following statements are correct? (Multiple choices)

A. Strengthen exercise, maintain a regular schedule, and improve one's own immunity

B. Wash hands correctly and in a timely manner

C. Increase indoor ventilation and keep the environment clean and hygienic

D. Adhere to safe eating practices and ensure meat is thoroughly cooked before consumption

E. Stay away from epidemic areas

F. Receive vaccination

G. Have a fixed sexual partner, avoid unprotected sexual behavior, and use condoms

35.What should you do if you suspect you have been infected with mpox?

A. Continue to work while sick

B. Hurry to flee to other "virus-free" areas

C. Voluntarily wear a mask and seek medical attention at the nearest designated hospital

D. Refuse medical treatment and endure it, assuming it will get better on its own

46.Regarding the matters to note when seeking medical treatment, which of the following statements is incorrect?

A. If you have symptoms such as fever or rash, you should seek medical attention in a timely manner

B. Voluntarily inform the doctor of your recent activity locations

C. Voluntarily inform the doctor if you have been in contact with suspected or confirmed cases

D. Conceal your recent activity routes and past medical history from the doctor

37.At present, there are anti-mpox virus drugs available in China.

A. Yes

B. No

C. Don't know

38.Vaccination against smallpox can prevent mpox.

A. Yes

B. No

C. Don't know

39.At present, the treatment of mpox in China mainly includes symptomatic supportive treatment and treatment for secondary bacterial infections.

A. Yes

B. No

C. Don't know

Survey on Attitudes Towards the Mpox Virus

40.mpox infection is a serious public health threat.

Strongly Disagree ○1 ○2 ○3 ○4 ○5 Strongly Agree

41.Based on the existing knowledge, skills and medical conditions, I believe that medical staff can diagnose, treat and manage mpox cases.

Strongly Disagree ○1 ○2 ○3 ○4 ○5 Strongly Agree

42.I am worried that mpox may become a global pandemic.

Strongly Disagree ○1 ○2 ○3 ○4 ○5 Strongly Agree

43.I am afraid that mpox will not be effectively controlled.

Strongly Disagree ○1 ○2 ○3 ○4 ○5 Strongly Agree

44.I am worried that mpox will add an extra burden to the national healthcare system.

Strongly Disagree ○1 ○2 ○3 ○4 ○5 Strongly Agree

45.I am worried about transmitting the mpox virus to my family.

Strongly Disagree ○1 ○2 ○3 ○4 ○5 Strongly Agree

46.If mpox were to become a pandemic in China, I believe the Chinese government could effectively control it.

Strongly Disagree ○1 ○2 ○3 ○4 ○5 Strongly Agree

47.As mpox cases spread, I need to learn more about the mpox disease.

Strongly Disagree ○1 ○2 ○3 ○4 ○5 Strongly Agree

48.I am afraid of contracting mpox myself.

Strongly Disagree ○1 ○2 ○3 ○4 ○5 Strongly Agree

49.If a mpox outbreak occurs, I am worried that it will lead to large-scale community lockdowns and management due to the epidemic.

Strongly Disagree ○1 ○2 ○3 ○4 ○5 Strongly Agree

50.I believe that mpox is preventable.

Strongly Disagree ○1 ○2 ○3 ○4 ○5 Strongly Agree

Survey on Behaviors Related to Mpox

51.Will you accept isolation if you are infected with mpox?

Strongly Disagree ○1 ○2 ○3 ○4 ○5 Strongly Agree

52.If a mpox outbreak occurs, as a medical student, how willing are you to actively cooperate with relevant epidemic response arrangements?

Strongly Disagree ○1 ○2 ○3 ○4 ○5 Strongly Agree

53.Regarding the mpox epidemic, have you proactively taken targeted preventive measures, such as wearing a mask, washing hands, etc.?

Strongly Disagree ○1 ○2 ○3 ○4 ○5 Strongly Agree

54.Regarding the mpox epidemic, have you proactively obtained or learned relevant knowledge about it?

Strongly Disagree ○1 ○2 ○3 ○4 ○5 Strongly Agree

55.Since the first indigenous mpox cases emerged in China, I have been paying close attention to information related to mpox.

Strongly Disagree ○1 ○2 ○3 ○4 ○5 Strongly Agree

Vaccination-related Behaviors

56.I am willing to receive the smallpox vaccine to prevent mpox infection.

Strongly Disagree ○1 ○2 ○3 ○4 ○5 Strongly Agree

57.If the mpox vaccine is provided free of charge, safely and effectively, I am willing to receive it to prevent mpox infection.

Strongly Disagree ○1 ○2 ○3 ○4 ○5 Strongly Agree

58.I am willing to pay for receiving the mpox vaccine.

Strongly Disagree ○1 ○2 ○3 ○4 ○5 Strongly Agree

59.If the Ministry of Health recommends it, I will receive the mpox vaccine.

Strongly Disagree ○1 ○2 ○3 ○4 ○5 Strongly Agree

60.I believe that receiving the mpox vaccine is a form of social responsibility.

Strongly Disagree ○1 ○2 ○3 ○4 ○5 Strongly Agree

61.I believe that the benefits of receiving the mpox vaccine outweigh its risks.

Strongly Disagree ○1 ○2 ○3 ○4 ○5 Strongly Agree

62.I believe that receiving the mpox vaccine can prevent mpox.

Strongly Disagree ○1 ○2 ○3 ○4 ○5 Strongly Agree

63.I believe that receiving the mpox vaccine can protect the health of myself and others.

Strongly Disagree ○1 ○2 ○3 ○4 ○5 Strongly Agree

64.I believe that receiving the mpox vaccine will reduce the risk of mpox infection and its complications.

Strongly Disagree ○1 ○2 ○3 ○4 ○5 Strongly Agree

65.I believe that receiving the mpox vaccine will help provide long-term immunity.

Strongly Disagree ○1 ○2 ○3 ○4 ○5 Strongly Agree

66.I believe it is necessary to receive the mpox vaccine to control mpox infections.

Strongly Disagree ○1 ○2 ○3 ○4 ○5 Strongly Agree

67.I am concerned about the potential side effects/adverse reactions of the mpox vaccine.

Strongly Disagree ○1 ○2 ○3 ○4 ○5 Strongly Agree

68.I am concerned about the effectiveness of the mpox vaccine.

Strongly Disagree ○1 ○2 ○3 ○4 ○5 Strongly Agree

69.I am concerned about the safety of the mpox vaccine.

Strongly Disagree ○1 ○2 ○3 ○4 ○5 Strongly Agree

70.I'm afraid of getting injections.

Strongly Disagree ○1 ○2 ○3 ○4 ○5 Strongly Agree

71.I am worried that receiving the mpox vaccine might cause me to contract mpox.

Strongly Disagree ○1 ○2 ○3 ○4 ○5 Strongly Agree

72.I will not get the mpox vaccine because this disease is not dangerous.

Strongly Disagree ○1 ○2 ○3 ○4 ○5 Strongly Agree

73.I don't need to get the mpox vaccine because my risk of contracting mpox is very low.

Strongly Disagree ○1 ○2 ○3 ○4 ○5 Strongly Agree

74.I believe that mpox vaccination should be mandatory to prevent mpox infections.

Strongly Disagree ○1 ○2 ○3 ○4 ○5 Strongly Agree

75.If the relevant administrative authorities require mandatory mpox vaccination, I will get the mpox vaccine.

Strongly Disagree ○1 ○2 ○3 ○4 ○5 Strongly Agree

76.If the mpox vaccine is approved, I will encourage my family and friends to get vaccinated against mpox.

Strongly Disagree ○1 ○2 ○3 ○4 ○5 Strongly Agree

77.I believe there is a lack of information regarding the effectiveness and safety of the mpox vaccine.

Strongly Disagree ○1 ○2 ○3 ○4 ○5 Strongly Agree

Willingness to work on the frontline during the mpox pandemic

78.If a mpox outbreak occurs, as a medical student, would you be willing to go to the frontline of the epidemic to support the work?

Strongly Disagree ○1 ○2 ○3 ○4 ○5 Strongly Agree
